# Discrete sequence production with and without a pause: the role of cortex, basal ganglia, and cerebellum

**DOI:** 10.3389/fnhum.2013.00492

**Published:** 2013-09-18

**Authors:** Anne-Lise Jouen, Willem B. Verwey, Jurjen van der Helden, Christian Scheiber, Remi Neveu, Peter F. Dominey, Jocelyne Ventre-Dominey

**Affiliations:** ^1^INSERM U846, Stem Cell and Brain Research InstituteBron, France; ^2^Department Cognitive Psychology and Ergonomics, University of TwenteEnschede, Netherlands; ^3^Radboud University Nijmegen, Donders Institute for BrainCognition and Behaviour, Netherlands; ^4^Center for Nuclear Imagery, HCLLyon, France; ^5^CNRS UMR5229, Center for Cognitive NeuroscienceBron, France

**Keywords:** cerebellum, basal ganglia, discrete sequence production task, motor skills, sequence learning

## Abstract

Our sensorimotor experience unfolds in sequences over time. We hypothesize that the processing of movement sequences with and without a temporal pause will recruit distinct but cooperating neural processes, including cortico-striatal and cortico-cerebellar networks. We thus, compare neural activity during sequence learning in the presence and absence of this pause. Young volunteer participants learned sensorimotor sequences using the discrete sequence production (DSP) task, with Pause, No-Pause, and Control sequences of four elements in an event related fMRI protocol. The No-Pause and Pause sequences involved a more complex sequential structure than the Control sequence, while the Pause sequences involved insertion of a temporal pause, relative to the No-Pause sequence. The Pause vs. No-Pause contrast revealed extensive fronto-parietal, striatal, thalamic and cerebellar activations, preferentially for the Pause sequences. ROI analysis indicated that the cerebellum displays an early activation that was attenuated over successive runs, and a significant preference for Pause sequences when compared with caudate. These data support the hypothesis that a cortico-cerebellar circuit plays a specific role in the initial processing of temporal structure, while the basal ganglia play a more general role in acquiring the serial response order of the sequence.

## Introduction

The discrete sequence production (DSP) task (Verwey, 1999) is a paradigm that is well suited to investigate sensorimotor sequence learning, and the effects of temporal pauses on sequence learning. In the DSP task, participants typically produce two novel, discrete sequences by responding to successive stimuli mapped to response keys in what is called a *reaction mode* of processing (Verwey, 2003). These sequences typically involve 2–6 elements. So, initially each stimulus must be processed to determine the correct response. With practice, subjects gradually change to producing these sequences in a *sequencing mode*, in which participants respond to the first stimulus by executing the entire keying sequence while taking little notice of later stimuli (Verwey, 2003; Verwey and Abrahamse, 2012). When temporal pauses are introduced in DSP sequences, subjects learn to incorporate these pauses into the sequence (Verwey, 1996; Verwey et al., 2009). Likewise, changing the placement of pauses in motor sequences can disrupt subjects' ability to recognize and perform sequences that they had previously learned (also see Stadler, 1993; Dominey, 1998a,b).

It is generally acknowledged that the basal ganglia are heavily involved in the sequential organization of motor sequences (Hikosaka et al., 1998). This is corroborated by studies with Parkinson patients, who have a basal ganglia deficit related to nigro-striatal dopaminergic dysfunction. These studies indicate that these patients have special difficulty performing later parts of movement sequences (e.g., Benecke et al., 1987a,b). Also, when executing two successive and different three-key sequences they were especially slow on the first element of the second sequence (Hayes et al., 1998). According to the model of Doyon et al. (2009), the striatum would be involved in motor sequence learning (i.e., the order of the movements) while the cerebellum would be involved in the initial identification and learning of the specific spatio-temporal structure of the sequence during motor adaptation, with a subsequent reduction in activity (also see, Penhune and Steele, 2012).

We have previously demonstrated that when a temporal pause is introduced in discrete sensorimotor sequences, subjects learn to incorporate these pauses into the sequence (Verwey, 1996; Verwey et al., 2009). The current research tests the hypothesis that processing sequences with a pause inserted at a fixed location will recruit neural processes that are distinct from those for processing related sequences with no pause (Doyon et al., 2009), with the striatum playing a more global role in sequence learning, and the cerebellum more involved in learning the spatio-temporal structure in the initial phase of learning, particularly in the presence of the pauses. To test this hypothesis we measured the blood oxygen level-dependent (BOLD) response using functional MRI while subjects performed the DSP task, learning novel, 4-element sequences, that were either *Pause* or *No-Pause*. The Pause sequence included a pause following the second response. In the No-Pause sequence such a pause did not occur.

## Methods

### Participants

Eighteen right-handed healthy volunteers participated in the study (mean age 22.5, *SD* = 1.8; 8 men). Handedness was determined by individual specification of the writing hand, a principal criteria in the Edinburgh inventory (Oldfield, 1971). The participants were all students from Lyon University. Prior to the scanning session, participants underwent an examination to validate their medical state and MRI compatibility. No participant had a history of neurological nor psychiatric disorders. All the participants completed the entire fMRI test, but two of them were discarded from the analysis because of the high number of motion related artifacts in the cerebral images. The protocol was approved by the Lyon Ethics Committee and the participants gave their informed consent before the scanning session.

### Task

**Figure 1:**
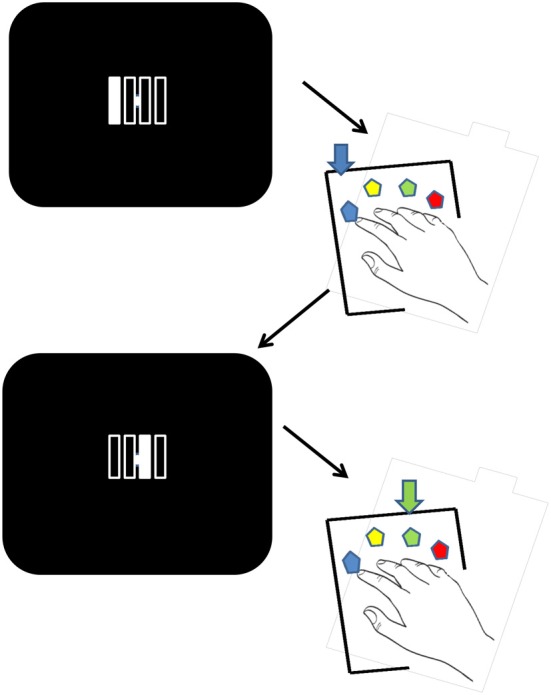
**Stimuli and experimental procedure**. Visual stimuli indicate the current key in the sequence. Each stimulus is made up of four unfilled vertical bars that are individually illuminated on each trial in the appropriate order. Each of the four bars corresponds to the spatially congruent key on the response pad. After a correct touch the next stimulus in the sequence is displayed. Faulty key presses were immediately followed by an error message (as a change from a white to a colored visual stimulus). The figure illustrates two stimuli and the corresponding responses.

The participants executed the DSP task (Verwey, 1999). They rested four fingers of the right hand on four keys of a key pad. The stimulus displayed on the screen involved filling one of four permanently displayed squares to which the participant responded by pressing the spatially corresponding key (Figure 1). As soon as the correct key had been pressed, the square was filled again with the background color and immediately [the response stimulus interval (RSI) was zero] another square was filled until four keys had been pressed. Participants were instructed to press the associated key as fast as possible while keeping errors to a minimum. Faulty key presses were immediately followed by an error message (by way of a change from a white to a colored visual stimulus). Key presses that anticipated the target during the pause were not counted as errors, and the sequence continued once the pause was complete.

### Stimuli

There were four (complex) experimental sequences: IRML, MLIR, RIML, and LMRI (Index, Middle, Ring, Little finger), and two (simple) control sequences: IMRL, LRMI. So, in the experimental sequences key presses were never carried out by adjacent fingers while the control sequences involved an order that is easy to learn, namely a left-to-right or a right-to-left succession of key presses.

These six sequences were split into a familiar and a novel set. Familiar sequences had been practiced the prior to the scanning (see Procedure below). Novel sequences were new to the subjects. Each set consisted of one control sequence and two experimental sequences. One of the experimental sequences in each set included a pause, a RSI of 800 ms between the second response (*R*_2_) and the third stimulus (*S*_3_) (all other RSIs were zero). No such pause occurred in the No-Pause and Control sequences.

Across all participants, the experimental sequences were balanced so that each of the four sequences occurred as frequently in each of the four experimental conditions (familiar vs. novel × Pause vs. No-Pause). Likewise, the two Control sequences were evenly distributed across the familiar and novel sequence sets.

### Procedure

The experiment included a practice session and the subsequent fMRI scanning session. Response times RT_1_–RT_4_ were measured in both sessions by the computer that presented the stimuli. In the practice session, participants sat in front of the computer display located on a table with the fingers of their right hand on a key pad. Practice involved 14 blocks, each including the three familiar sequences (Pause, No-Pause, and Control) in a random order. The entire training session included 1500 trials (~500 trials with each sequence) and lasted ~2 h 30 min. A 10 min pause was inserted at the middle of the training session.

In the scanning session, which was preceded by a 30 min break after the practice session, the participant was comfortably installed in the MRI scanner. Head movements were prevented using a foam cushion and a frontal band that were attached to the scanner bed. The visual stimuli (see Figure 1) were displayed by video-projector on a translucent screen located behind the scanning bay. The participant looked at the screen via a mirror fixed inside the scanner at 20 cm over the participant's head. The key pad was located comfortably on the participant's lap.

The scanning session involved 4 runs, two with the three familiar and two with the three novel sequences. Half the participants started with a familiar sequences run, the other half with a novel sequences run. Then familiar and novel runs alternated. Each run lasted ~10 min and successive runs were separated by 2 min breaks. Each run included 150 trials with the three sequences in random order (i.e., 50 of each sequence in each run). The interval between the first stimulus of two successive sequences was 4 s, with occasional trials with an 8 s interval, to help ensure that activation signals for different conditions did not overlap. fMRI data was acquired with a 1.5T system (Siemens CTI) at the Imaging Center of Lyon (CERMEP “Imagerie du vivant”).

Brain scans involved assessment of the BOLD fMRI signal. The anatomical images of the brain (3D MPR) were recorded using a T1-weighted sequence. For each run, whole brain coverage was obtained with Echo Planar Imaging (EPI) images (repetition time *TR* = 2500 ms, echo time *TE* = 60 ms, and flip angle 90°). Twenty-six brain sections were acquired in an interlaced mode parallel to the AC-PC plane. Slices had a thickness of 4.4 mm [matrix 64 × 64; and field of view (FOV) = 230 mm, voxel size, 3.4 × 3.4 × 4 mm]. Following functional image acquisition, a high-resolution T1-weighted anatomical image was acquired (*TR* = 1880 ms; *TE* = 3.93; lip angle 15°; matrix 256 × 256; and slice thickness 1 mm).

### Data analysis

The focus of the current analysis is on the effects of the temporal pause in DSP learning. Analyzes comparing data from the familiar sequences with and without the pause will be presented later in a separate paper. Thus, in order to examine learning in the presence vs. absence of the temporal pause, we focus on results for the set of novel sequences, which included the Pause sequence (with the 800 ms RSI separating R_2_ and S_3_), and the No-Pause and Control sequences. The response time (RT) analysis involved an analysis of variance (ANOVA) using a three-way repeated measures design, with factors RUN (2) × PAUSE (3—No-Pause, Pause, Control) × KEY (4).

Preprocessing of fMRI data was conducted using SPM 2. For the fMRI analysis, the first five scans of each run (i.e., the first 12.5 s and hence the first two or three sequences) were discarded to eliminate non equilibrium effects of magnetization and warming up effects in the participants. For preprocessing, the functional images were realigned with respect to the first functional image and were corrected for slice acquisition timing in reference to the middle slice in each scan. The resulting volumes were spatially normalized to fit to an EPI template in Montreal Neurological Institute (MNI) space, with 2 × 2 × 2 mm voxels. The normalized images were then spatially smoothed using an isotropic Gaussian filter kernel at an 8 Hz bandwidth. For each participant the BOLD impulse response to different event types were modeled in the context of the general linear model (GLM) by using the hemodynamic response function (HRF) convolved with a delta (event-related) function.

Processing of the fMRI data involved the Statistical Parametric Mapping software (SPM 2, Wellcome Department of Imaging Neuroscience, London UK; http://www.fil.ion.ucl.ac.uk/spm) running under Matlab (The Mathworks, Inc., Natick, StateMA, USA). On the basis of the GLM model (Friston et al., 1994), the task related BOLD changes were estimated as linear combinations of the individual regressors and stored as participant specific contrast images. Several contrasts were realized using the different regressors in order to study the differences between the three sequences (Pause = P, No-Pause = N, Control = C) i.e., P > C, N > C, P > N, and N > P. These contrasts were selected to extract the activated neural structures specifically involved in processing sequences with and without the imposed pause.

For the statistical group analysis, the individual contrast images were then processed in a second–level random effects model by using a one sample *t*-test model to extract significant neural activations. Significance level was established at a false discovery rate (FDR) threshold of *p* < 0.05 corrected for whole-brain voxels with minimal spatial extent of 15 contiguous voxels per cluster. To determine the neural structures activated in common during the Pause and No-Pause sequences, we performed a conjunction analysis based on Nichols' procedure (Nichols et al., 2004) for the two Pause vs. Control and No-Pause vs. Control contrasts (*p* uncorrected < 0.001 for multiple comparisons). All MNI coordinates of the cerebral activation foci were transformed into Talairach coordinates using the formula developed by Matthew Brett (http://imaging.mrc-cbu.cam.ac.uk/imaging/MniTalairach), and Brodman areas were determined using the stereotaxic atlas (Talairach and Tournoux, 1988).

By using the Marsbar toolbox (http://marsbar.sourceforge.net), regions-of-interest (ROIs) were built from the “peak” activation coordinates of each relevant cluster as a 10 mm radius cubic 3-D volume. For each ROI, we evaluated the percentage of signal change from “beta” values (slope of the regression line for each regressor) for that ROI. The estimated changes of activity were analyzed by a repeated measures ANOVA, including the within-subject factors Sequence (Pause, No-Pause, Control), Hemisphere (right vs. left), and Run (first vs. second). *Post-hoc* comparisons were performed with a Scheffé *post-hoc* analysis.

### Apparatus

The experimental protocol was implemented in Presentation (Neurobehavioral Systems, Albany, USA) on a Windows XP based PC. In the DSP task, a Luminar key pad (Cedrus, USA) that is suited for used in MRI scanners was used for registering key presses. The reaction time data were analyzed using Statistica (Statsoft Inc.).

## Results

### Behavioral results

**Figure 2:**
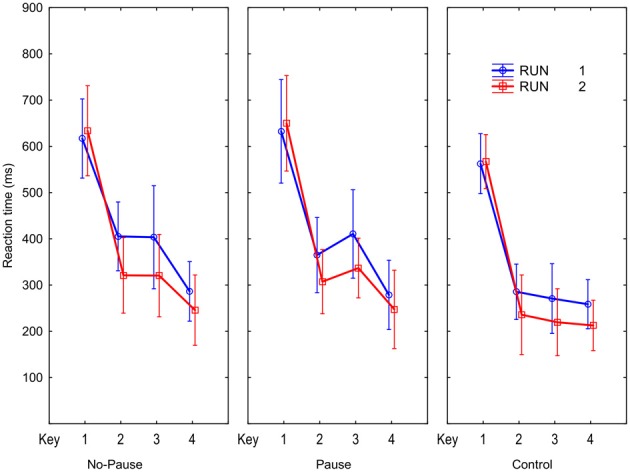
**Reaction times for the four key presses in the No-Pause, Pause, and Control conditions for the two Novel sequence runs during scanning**.

Figure 2 illustrates the RTs obtained while the novel sequences were carried out during scanning. We recall that each sequence has a unique order that is signaled to the subject by the first key, so while the first key is not predictable by learning, keys 2–4 are predictable and their RTs should display learning effects. We observe that RTs are clearly reduced from Run 1 to Run 2, for keys 2–4, and not key 1. The Run main effect, *F*_(1, 15)_ = 15.4, *p* < 0.01, confirmed that RTs are reduced across the two successive runs. The Run × Key interaction, *F*_(3, 45)_ = 17.5, *p* < 0.001, confirms that this was caused exclusively by learning the predictable responses (RTs 2–4 reduced 57 ms), *F*_(1, 15)_ = 29.6, *p* < 0.001, and not by simple preparation speedup RT1, *F*_(1, 15)_ < 1, *p* > 0.05. Thus, the execution rate of all three sequences improved to a similar degree while preparation did not improve. The Run × Sequence interaction [*F*_(2, 30)_ = 1.5, *p* = 0.2] indicates that the improvement from Run 1 to Run 2 does not vary as a function of sequence type. This confirms significant and equivalent learning of the novel Pause and No-Pause sequences.

The results in Figure 2 indicate the pause before key 3 did not produce an increased RT in the Pause sequences. *Post-hoc* comparisons revealed that RTs for individual keys, including key 3, of the Pause and No-Pause sequences did not differ. The similarity of the RTs for key 3 in the Pause and No-Pause sequences suggest that in the Pause sequence participants quickly learned to anticipate when S_3_ would occur.

An ANOVA with the same design on arcsine transformed error proportions showed that more errors were made in the Pause (1.8% per key) than in the No-Pause and Control (1.1, 1.0% per key, respectively) sequences, *F*_(2, 30)_ = 5.1, *p* < 0.05, and error rate differed across the 4 keys, *F*_(3, 45)_ = 10.3, *p* < 0.001. Planned comparison following the Sequence × Key interaction, *F*_(6, 90)_ = 3.7, *p* < 0.01, showed that more errors were made on R_3_ in the Pause sequence relative to the other sequences (3.4 vs. < 2%), *F*_(1, 15)_ = 24.0, *p* < 0.001. Thus, while anticipating onset of S_3_ in the Pause sequence was learned rapidly, the actual response at the third position was somewhat more difficult to learn in the Pause than in the No-Pause sequence.

### fMRI results

#### Sequence formation activation by conjunction analysis

We determined the cerebral sites activated in common during the execution and learning of the Pause and No-Pause sequences, relative to the Control sequence, by using a conjunction analysis. To that end, we analyzed the conjunction of Pause minus Control (P > C), and No-Pause minus Control (N > C) contrasts (at *p* uncorrected < 0.001), in order to identify the common regions activated when learning a new sequence independently of the temporal pause (Table 1). The common neural substrates of developing a motor sequence representation—relative to the control sequence—are revealed as an antero-posterior network with a major bilateral temporo-prefrontal activation. In the superior temporal cortex, the activation formed a two-fold pattern with a large activation including BA42/22 and a smaller and more posterior activation in BA22. In the precentral gyrus, we identified bilaterally two activation clusters at the surface and in the depth of the precentral gyrus in BA6. A more extended pattern was identified bilaterally in the anterior cingulate cortex (24/32) and in the prefrontal cortex including the inferior and middle gyri BA (BA47/11) on the left. A small BOLD change was found in the left inferior parietal lobe BA7.

**Table 1:** **Common areas obtained by conjunction analysis of No-pause vs. Control (N > C) and Pause vs. Control (P > C) contrasts (p uncorrected < 0.01)**.

**Anatomical area**	**BA**	***x*,**	***y*,**	***z***	***t*-stat**	**Ke**
R Superior temporal gyrus	22/42	69,	−25,	12	4.76	139
		69,	−23,	3	3.99	
R Superior temporal gyrus	22	36,	−44,	15	3.72	21
L Inferior parietal gyrus	7	−40,	−64,	42	3.07	21
L Precentral	6	−50,	−5,	22	3.65	16
R Precentral	6	40,	−4,	30	3.80	45
		28,	1,	26	3.00	
L Middle frontal gyrus	11	−37,	36,	−18	4.15	85
L Inferior frontal gyrus	47	−50,	40,	−12	3.74	
R Cingulate	23/24	26,	−16,	32	4.19	141
		32,	−29,	29	3.61	
L Cingulate	24/32	−30,	15,	25	4.09	73
		−22,	17,	32	3.41	
R Cerebellum—Dentate		15,	−59,	−19	4.28	87
		22,	−61,	−20	3.23	
		26,	−69,	−20	3.22	
R Cerebellum—Culmen		4,	−55,	−6	4.02	28
L Cerebellum—Declive		−30,	−59,	−16	3.88	51
		−24,	−67,	−17	2.80	
R Caudate		14,	5,	15	3.67	87

Ke—cluster size in voxels.

The conjunction analysis in Table 1 revealed activity in two principle subcortical structures: (1) the cerebellum, as substantial clusters in the right culmen and dentate nucleus and in the left declive of the cerebellum, and (2) the basal ganglia, forming a significant activation in the body of caudate nucleus, lateral part.

In summary, sequence formation in the Pause and No-Pause sequences together, relative to the Control sequence, implicated a large bilateral cortico-subcortical network including temporo-prefrontal cortex, the cerebellum and basal ganglia preferentially. A summary visualization of this sequence formation network is presented below.

#### Pause vs. No-pause sequence contrast analysis

**Figure 3:**
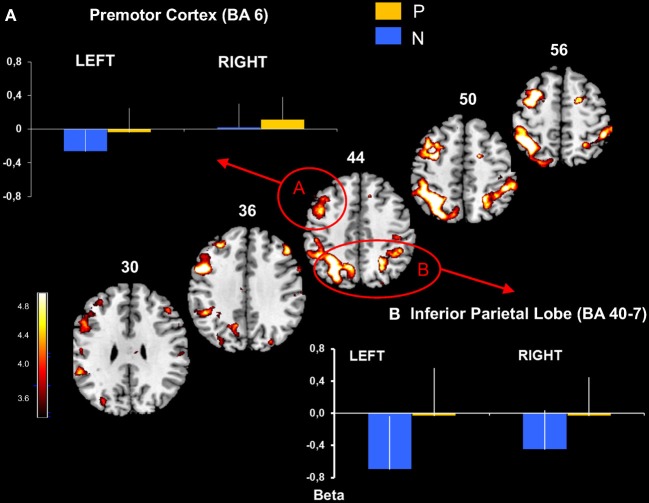
**Premotor and inferior parietal cortical activation obtained with P > N contrast**. Activated prefrontal regions shown on template axial sections including the left premotor cortex, BA 6 **(A)** and bilaterally the inferior parietal cortex, BA 40 and 7 **(B)**. The graphs represent mean beta values extracted at the maximal peak of activation during No-Pause (N) and Pause sequences (P) in prefrontal clusters. *P* corrected < 0.01. Bars = standard deviations.

**Figure 4:**
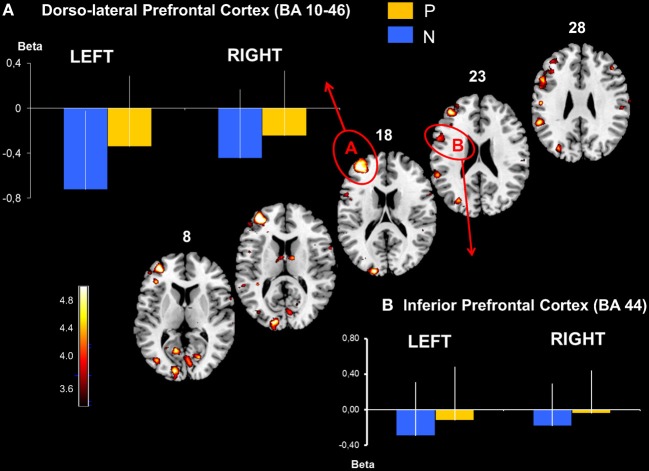
**Dorso-lateral prefrontal and inferior prefrontal cortical activation obtained with P > N contrast**. Activated prefrontal regions shown on template axial sections including the left hemisphere the dorsolateral prefrontal cortex, BA 46-10 **(A)** and the inferior prefrontal cortex, BA 44 **(B)**. The graphs represent mean beta values extracted at the maximal peak of activation during No-Pause (N) and Pause sequences (P) in the prefrontal clusters. *P* corrected < 0.01. Bars = standard deviations.

**Figure 5:**
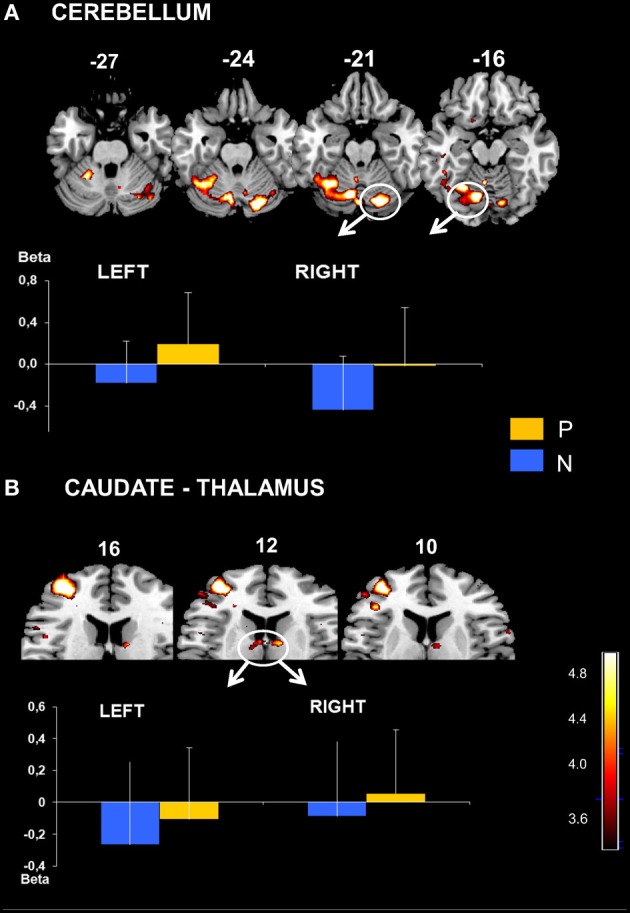
**(A)** Cerebellar activation obtained with P > N contrast. Bilateral activation in cerebellum displayed on the template axial sections. The graph represents mean beta values extracted at the maximal peak of activation during No-Pause (N) and Pause sequences (P) in right and left cerebellum clusters (indicated in the Figure). *P* corrected < 0.01. Bars = standard deviations. Caudate and Thalamic activation. **(B)** Bilateral activation in caudate nucleus displayed on template axial sections. The graph represents mean beta values extracted at the maximal peak of activation during No-Pause (N) and Pause sequences (P) in right and left caudate clusters. *P* corrected < 0.05. Bars = standard deviations.

**Figure 6:**
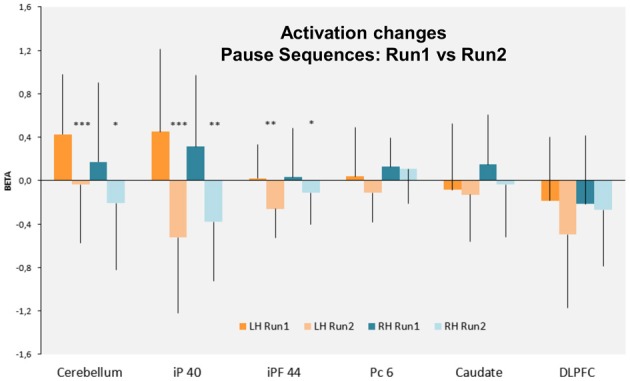
**Bars graph indicating the rCBF changes (Beta) in the cortical and subcortical ROIs defined in the left and right hemispheres (LH and RH, respectively)**. Note that only the cerebellum and the inferior parietal cortex BA40 (iP 40) and to a less extent the inferior prefrontal cortex BA 44, are the most activated bilaterally during run 1 and significantly decrease their activity during run 2. Bars: standard deviation. iP 40: inferior parietal cortex BA40—iPF44: inferior prefrontal cortex BA44—Pc6: precentral cortex BA6—DLPFC. ^***^p < 0.001; ^**^p < 0.01; ^*^p < 0.05.

In order to examine the effect of processing the pause we examined the contrast Pause > No-Pause (P > N), revealing the effect of the pause on BOLD changes (Table 2 and Figures 3–6).

**Table 2:** **Specification of regions with significant activation in the Pause vs. No-pause contrast (P > N) at *p* < 0.01 (FDR) and *p* < 0.05 (FDR) and in the No-pause vs. Pause contrast (N > P) at *p* < 0.001 (uncorrected)**.

**Anatomical area**	**BA**	***x,***	***y,***	***z***	***t*-stat**	**Ke**
**PAUSE > NO-PAUSE ***p*** < **0.01** (FDR)**							
L Inferior parietal lobule^*^	40	**−38,**	**−46,**	**56**	9.25	621
		−32,	−50,	49	7.87	
	7	−26,	−60,	44	6.88	
R Postcentral gyrus	2	48,	−32,	62	8.33	110
R Inferior parietal lobule^*^	40	**48,**	**−36**	**48**	6.25	
R Inferior parietal lobule	40−7	24,	−50,	45	6.34	28
		26,	−60,	44	5.23	
L Middle prefrontal gyrus^*^	6	**−28,**	**10,**	**49**	7.9	157
		−34,	5,	55	6.72	
		−30,	2,	46	6.5	
L Middle prefrontal gyrus^*^	46−10	**−34,**	**49,**	**7**	7.12	117
		−30,	45,	12	6.44	
		−38,	45,	16	5.88	
L Middle prefrontal gyrus^*^	9−44	**−53,**	**9,**	**33**	6.44	61
		−46,	9,	35	5.96	
		−42,	8,	42	5.13	
L Cerebellum—Declive^*^		**−10,**	**−67,**	**−13**	7.43	76
L Cerebellum—Declive		−26,	−57,	−11	7.02	45
		−34,	−55,	−17	5.36	
R Cerebellum—Declive^*^		**14,**	**−74,**	**−13**	6.71	67
		20,	−79,	−16	6.01	
		26,	−73,	−18	5.33	
**PAUSE > NO-PAUSE ***p*** < **0.05** (FDR)**							
L and R Caudate^*^		**14,**	**−3,**	**15**	4.26	175
L Thalamus		−4,	−1,	11	4.68	
**NO PAUSE > PAUSE ***p*** < **0.001** (uncorrected)**							
R Superior prefrontal gyrus	8	18,	49,	44	57	5.47

* ROIs used for statistical analysis. Ke—cluster size in voxels.

This revealed an extended network of cerebral activation spreading from the parietal cortex to the prefrontal cortex. These BOLD changes formed 4 principle cortical activation loci, predominantly in the left hemisphere at *p* corrected (FDR) < 0.01.

The parietal activation was bilateral with the largest cluster located in the left hemisphere including BAs 7 and 40 in the inferior and the superior parietal lobes (Figure 3). In the prefrontal cortex, significant activation was found in 3 principle regions: (1) a caudal locus focused in the middle prefrontal gyrus BA6 (Figure 3); (2) ventrally a smaller locus in BA 44 of the middle frontal gyrus; and (3) the most rostral activation extending from the middle to the inferior frontal gyrus in BAs 46 and 10 (Figure 4). Significant subcortical activation was identified bilaterally in the cerebellum declive forming a set of postero-dorsal clusters (Figure 5).

Significant BOLD change was observed in the midbrain in the thalamus and in the body of caudate nucleus with a voxel threshold at *p* corrected (FDR) < 0.05 (Table 2 and Figure 6). At this *p* level, the clusters of the antero-posterior cortical network activation remained in the same location but were enlarged. Contrasting the No-Pause and Pause (N > P) conditions revealed a small focus of activation identified at *p* < 0.001 uncorrected in the right superior frontal gyrus BA8.

In summary, the P > N contrast revealed significant activation in the parietal lobe (BA 7/40), in the frontal lobe (left middle prefrontal cortex, BA6, 44/9, and BA 10/46), in the cerebellum (declive), and in the midbrain (thalamus and caudate). In contrast, the N > P contrast was associated with significant activity in the frontal cortex (superior frontal gyrus BA 8).

#### Specific cerebral activation by regions of interest analysis

On the basis of the voxel based analysis of the P > N activations we defined six significant (*p* < 0.01, FDR: corrected for multiple comparisons) ROIs (Table 2). These were identified in the cerebellum (CB), the parietal cortex (P-BA 40, 7), the premotor cortex (PreM-BA 6), the dorsolateral prefrontal cortex (DLPFC-BA 46, 10) and the middle prefrontal gyrus (BA 44). Activations in the caudate nucleus (C) were also found, at a lower threshold (FDR corr, *p* < 0.05). As illustrated in Figures 3, 4, direct comparisons of rCBF changes (beta values) between the different sequence conditions (main Sequence effect) demonstrated significant pause related activation bilaterally in the inferior parietal cortex [Left: *F*_(2, 30)_ = 32 and Right: *F*_(2, 30)_ = 16; *p* < 0.001] and only in the left hemisphere for the premotor cortex [*F*_(2, 30)_ = 10, *p* < 0.001], the DLPFC [*F*_(2, 30)_ = 12, *p* < 0.001], the inferior prefrontal cortex [*F*_(2, 30)_ = 18, *p* < 0.001]. In the subcortical structures (Figures 5, 6), a main Sequences effect was found bilaterally in the cerebellar declive [Left: *F*_(2, 30)_ = 16 and Right: *F*_(2, 30)_ = 12; *p* < 0.001] and in the caudate nucleus [Left and Right: *F*_(2, 30)_ = 14, *p* < 0.001]. As these ROIs were defined based on the P vs. NP contrast, these results are expected.

In contrast, the RUN effect on rCBF changes was restricted to few regions: in the parietal cortex [Left: *F*_(1, 15)_ = 7, *p* < 0.02, Right: *F*_(1, 15)_ = 16, *p* < 0.001], in the left inferior prefrontal cortex [*F*_(1, 15)_ = 7, *p* < 0.02] and in the cerebellar declive [Left: *F*_(1, 15)_ = 14, *p* < 0.001, Right: *F*_(1, 15)_ = 12, *p* < 0.001]. *Post-hoc* comparison (Scheffé) revealed a pattern of learning related activity (i.e., Run1 vs. Run2) that was differentiated between a parieto-cerebellar network and prefrontal cortex. In both cerebellum and parietal cortex ROIs, BOLD activity decreased between the 2 runs for Pause (*p* < 0.001) and No-Pause (*p* < 0.01) sequences. In contrast, in prefrontal ROI (BA9-44), we observed a Sequence × Run interaction [*F*_(1, 15)_ = 4.6, *p* < 0.05] demonstrating a significant Run1 vs. Run2 reduction for the Pause (*p* < 0.001) but not the No-Pause sequence. No effects of the run were found in the other prefrontal ROIs and in the caudate nucleus. Figure 6 shows the effect of the runs on the rCBF changes of these ROIs.

Overall, the contrast and ROI analyzes demonstrate that (1) three regions (cerebellum, parietal cortex, and inferior prefrontal cortex) display a significantly increased activation for the Pause sequences. (2) In two of these regions (cerebellar and parietal ROIs), there is a significant and equivalent reduction in activation for both Pause and No-Pause sequences in the transition from Run 1 to Run 2 where sequence learning occurs. (3) In the prefrontal cortex, this reduction from Run 1 to Run 2 is only observed for the Pause sequences, with equal activation for both sequence types in Run 2.

**Figure 7:**
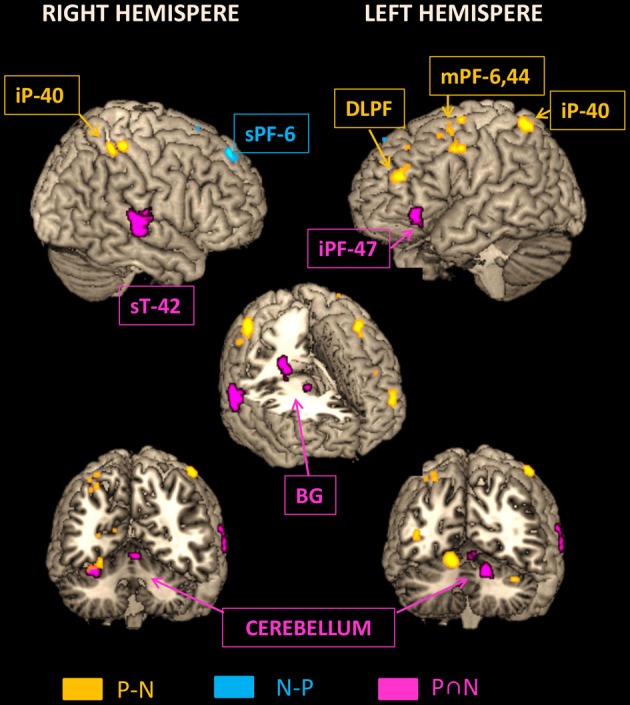
**Representation on rendered templates of the activated networks involved in the sequence formation (pink: N AND P conjunction), in the temporally Pause sequence (yellow: P > N), and in the temporally No-Pause sequence (blue: N > P)**. The cutouts (three lower images) display the activated structures in the depth of cortical gyri and in the sub-cortical structures, including basal ganglial. iP, Inferior parietal cortex; sT, Superior temporal cortex; Pc, Precentral cortex; sPF, Superior prefrontal cortex; DLPF, Dorso-lateral prefrontal cortex; iPF, Inferior prefrontal cortex; mPF, Middle prefrontal cortex; BG, Basal ganglia; Cb, Cerebellum.

Figure 7 illustrates on lateral 3D views of the brain the neural structures underlying (1) the sequence formation network identified by conjunction analysis, (2) the temporal integration in Pause sequence by ROIs analysis of Pause vs. No-Pause sequences and (3) the activated prefrontal area BA8 identified by No-Pause vs. Pause sequences.

## Discussion

The organization of our sensorimotor experience is typically not uniform in time (Allen and Ferguson, 1994; Dominey, 1998a,b; Dominey and Ramus, 2000; Ventre-Dominey et al., 2002; Shin and Ivry, 2003; Bengtsson et al., 2004). In the current study we investigated brain mechanisms for the learning of sequential behavior in the situation that a temporal pause is introduced into the sequence.

### General learning effects in the discrete sequence processing task

Given that the present study is one of the first to examine the development of fixed sequences of limited length, that are typical for the DSP task, we first consider the conjunction analysis for the activation common to the Pause and No-Pause sequences relative to a Control sequence requiring little serial control. This analysis revealed an extended cortical and subcortical network for sequence learning that included precuneus (left BA7), anterior cingulate and premotor cortices (bilateral BA6). These regions are known to participate in finger sequence learning (Harrington et al., 2000; Jenkins et al., 2000; Haaland et al., 2004; Halsband and Lange, 2006). Activation in the superior temporal gyrus (BA22/42) as well as the inferior frontal gyrus (BA47) was also identified in this common sequence formation process. These areas have been implicated in implicit finger sequence learning in a serial reaction time task too, in which successive items were presented for 1 s during which the subjects were to respond with a keypress (Daselaar et al., 2003). Interestingly, activation in BA22 in a finger tapping task has also been associated with finger-specific activation (Aoki et al., 2005). Likewise, BA47 is activated in rhythmic tapping tasks (Vuust et al., 2011). In addition to these cortical sites, we observed a subcortical activation involving cerebellum and basal ganglia whose implications have been previously described in sequence formation. In this context, the cerebellum would be involved in optimizing sensory information and monitoring the motor output while basal ganglia-cortical loops would be involved in movement selection with sequence learning (Jueptner and Weiller, 1998). Accordingly, the co-activation pattern of cerebellum and basal ganglia has been described in explicit and implicit sequence learning with a cerebellar decrease as sequence learning proceeds and a fronto-striatal activation maintenance during the learning plateau (Grafton et al., 1995; Rauch et al., 1995, 1997; Doyon et al., 1996, 2009; Doyon, 2008). In short, the initial practice of new, short fixed sequences is associated with enhanced activation—relatively to a simple Control sequence—in a network involving frontal areas (bilateral premotor cortex BA6 and anterior cingulate cortex- left orbitofrontal cortex BA11/47), associative visuomotor areas (left precuneus cortex BA7), temporal areas (right secondary auditory cortex BA22/42), and in the cerebellum and right caudate.

### Processing of pause and no-pause sequences

We now consider the specific effects Pause vs. No-Pause sequences. Behavioral analyzes showed that for the Pause and No-Pause sequences, learning was demonstrated as their execution times improved over the two experimental runs. The similarity between the RTs of the Pause and No-Pause sequences demonstrates that participants learned the two sequences. It is well documented that the insertion of such pauses produces a reliable and specific effect on learning, which is to induce a segmentation of the sequence at that location (Verwey and Eikelboom, 2003; Verwey et al., 2009). Likewise, we have clear evidence that repeated presentation of pauses at specific locations in sequences leads to an integration of these pauses into the learned representation, as the expression of learning is significantly impaired when the pause structure is modified during testing (Dominey, 1998a,b).

It is worth noting that in contrast, related studies of sequence learning have shown that when pauses are inserted randomly, sequence learning is disrupted and even eliminated. Stadler (1995) demonstrated that when the response-to-stimulus interval is increased on a random selection of half of the trials from 400 to 2000 ms there is a significant disruption of sequence learning, with learning effects reduced by more than 50% (from 227 to 97 ms for simple sequences, and 74 to 21 ms for more complex sequences). Similarly, Deffains et al. (2011) demonstrated impaired sequence learning in the non-human primate when all elements of the sequence were separated by long pauses. Our observation of equal learning for the Pause and No-Pause sequences indicates that the pause was likely integrated within the sequence structure and learned, and was not processed as a temporal disruption of the sequence, thus, distinct from the perturbing effects of random pauses demonstrated by Stadler (1995) and Deffains et al. (2011).

Learning the Pause and No-Pause sequences was associated with a distributed network including bilateral cerebellar-parietal circuits as well as left fronto-striatal circuits, including caudate. In the case of our Pause sequences, the system must accommodate both the linear order of the sequence elements and the integration of the pause. These dimensions correspond to the Pause factor, and the progression of learning over successive runs, i.e., the Run factor. Interestingly, we observed interactions between sequence types and temporal learning process when comparing the different sites of activation. As learning progresses, the cerebellar, parietal and prefrontal activation displays a significant reduction of activation for the Pause sequences (see Figure 6). For the prefrontal cortex, this reduction results in equivalent activation for Pause and No-Pause sequences in Run 2. Combined with the observation that significant learning took place for the Pause sequences, this indicates that the pause was learned. This suggests that the effects observed for Pause sequences are not simply due to the non-specific effects of the presence of the pause, but rather, it is linked to the integration of the pause within the sequence. Future research could address this question by examining neural and behavioral responses to randomly inserted pauses.

### Neural substrates of serial and temporal processing

The comparison of caudate and cerebellum activity revealed the potential existence of dissociated systems for different structural dimensions of sequence learning. Both cerebellum and caudate display increased activation for the Pause vs. No-Pause sequences. In the progression from Run 1 to Run 2, the cerebellar activation is reduced, while the caudate displays no effect of Run. This suggests that the temporal integration process may take place in two domains. One requires the identification and calibration of the temporal structure, and the second implies the integration of this structure into the holistic representation of the sensorimotor sequence. The first process may be reflected by cerebellar activation, with a greater activation for Pause sequences, and a reduction in activation from Run 1 to Run 2. The second process likely corresponds to caudate activation, with a greater activation for Pause sequences that is maintained from Run 1 to Run 2. Interestingly, at the level of the cerebral cortex, while the activity in the inferior parietal cortex bilaterally formed a similar pattern as the cerebellum, the left inferior prefrontal cortex activity changed as the learning progressed only for the Pause sequences. Indeed, the prefrontal area BA44 displayed a Pause × Run interaction, with a significant reduction in activation from Run 1 to Run 2 for Pause but not No-Pause sequences. This is consistent with findings that a cerebellar and prefrontal circuit may be involved in the discrimination of temporal information (Mathiak et al., 2004). These data support the hypothesis that a cortico-cerebellar circuit plays a specific role in the initial processing of temporal structure, while the basal ganglia play a more general role in acquiring the serial response order of the sequence.

Our Pause sequences activated, relative to the No-Pause sequences, predominantly the left sensori-motor cortical network including the premotor cortex and DLPFC. By comparing heterogeneous vs. simple, repetitive sequences, Haaland et al. (2004) reported that complex finger sequences preferentially engaged parietal cortex and cerebellum. Moreover, they described a relationship between the asymmetry of the sensorimotor cortical activation and the sequence complexity. It is worth noting that in our study the insertion of the pause in the Pause sequences can be considered as a complexity increase compared to the No-Pause sequences. In agreement with Haaland et al.'s findings (2004), our Pause sequences activated, relative to the No-Pause sequence, predominantly the left sensori-motor cortical network including the premotor cortex and DLPFC which might be implicated in advanced planning and abstract organization of complex motor sequences. These results are consistent with a number of related studies, while extending them based on the analysis of Pause vs. No-Pause sequences. The cerebellum and parietal cortex have been implicated in sensorimotor sequence learning (Hikosaka et al., 2002), particularly in the early stages of learning (Rauch et al., 1997; Doyon and Ungerleider, 2002; Lehericy et al., 2005; Halsband and Lange, 2006; Doyon et al., 2009). For example, in an fMRI study of motor sequence learning, Doyon and Ungerleider (2002) demonstrate that cerebellum as well as inferior parietal and dorsal premotor cortices diminished their activity at the stable learning stage while the striatum activity coupled to anterior motor cortical areas is maintained, suggesting a role of basal ganglia in storage and automatization of the motor performance. Similarly, neurophysiological changes have been subsequently observed by Lehericy et al. (2005) during early and advanced motor learning. Compared to these studies, we provide new insights in the feedback control role of the cerebellum in early sequence learning, as the bilateral cerebello-parietal circuits might also contribute to the temporal integration and sequence segmentation of initial learning. In the context of temporal structure, Sakai et al. (1999, 2004) demonstrated cerebellar posterior lobe activation in the processing of non-metrical (vs. metrical) rhythm, which would correspond to the insertion of the pause in our sequences. Indeed, these authors point out that the posterior cerebellum has been implicated in explicit temporal representation. In a related study, Sakai et al. (2002) examine the learning of serial order vs. temporal structure and observe prefrontal, premotor, inferior parietal cortical and cerebellar activation when both are learned. Interestingly, when timing alone was learned, activation was restricted to areas including parietal cortex and cerebellum, while sequence learning in the absence of temporal structure eliminated the cerebellar activation. This is consistent with our interpretation that posterior cerebellar (declive) activation reflects that subjects are integrating the temporal structure of the pause, and that as the pause is integrated the effect is reduced as the sequence learning progresses (Run 2). Learning the larger time scale structure of the entire sequence is reflected by the striatal activity that is less influenced by run. In the context of such a distinction, it has been suggested that the cerebellum is involved in processing of temporal structure at a timescale of tens to hundreds of milliseconds, while the basal ganglia would be involved more at a timescale of seconds (Mauk and Buonomano, 2004).

Further examining this distinction, Shin and Ivry (2003) studied concurrent temporal and spatial sequence learning in patients with Parkinson's disease, and with cerebellar lesions. They observed that while PD patients were capable of learning both serial and temporal structure, they failed to integrate the two into a unified representation, as would be required in our task, where the temporal pause must be integrated into the spatial sequence. The cerebellar lesion patients displayed a global sequence learning deficit. In addition, we previously demonstrated in Parkinson patients a dissociation between reaction time processing implicating the striatum in impaired stimulus-response timing adaptation vs. a preserved frequency timing processing involving other structures than striatum, likely the cerebellum (Ventre-Dominey et al., 2002).

## Conclusion

In summary, we interpret these results in the context whereby both the striatum and cerebellum preferentially intervene in processing of the pause. Over the course of the learning, the cerebellar activation is reduced. In parallel with this, the caudate continues to participate in the integration of the pause within the sequence over the duration of the two runs. These processes make up part of the neural system that exploits natural spatio-temporal organization of sensorimotor sequences.

### Conflict of interest statement

The authors declare that the research was conducted in the absence of any commercial or financial relationships that could be construed as a potential conflict of interest.

## References

[B1] Allen J. Ferguson G. (1994). Actions and events in interval temporal logic. J. Logic Comput. 4, 531 10.1093/logcom/4.5.531

[B2] Aoki T. Tsuda H. Takasawa M. Osaki Y. Oku N. Hatazawa J. (2005). The effect of tapping finger and mode differences on cortical and subcortical activities: a PET study. Exp. Brain Res. 160, 375–383 10.1007/s00221-004-2008-9 15368088

[B3] Benecke R. Rothwell J. C. Dick J. P. Day B. L. Marsden C. D. (1987a). Disturbance of sequential movements in patients with Parkinson's disease. Brain 110, 361–379 10.1093/brain/110.2.361 3567527

[B4] Benecke R. Rothwell J. C. Dick J. P. Day B. L. Marsden C. D. (1987b). Simple and complex movements off and on treatment in patients with Parkinson's disease. J. Neurol. Neurosurg. Psychiatry 50, 296–303 10.1136/jnnp.50.3.296 3559611 PMC1031794

[B5] Bengtsson S. L. Ehrsson H. H. Forssberg H. Ullen F. (2004). Dissociating brain regions controlling the temporal and ordinal structure of learned movement sequences. Eur. J. Neurosci. 19, 2591–2602 10.1111/j.0953-816X.2004.03269.x 15128413

[B6] Daselaar S. M. Rombouts S. A. Veltman D. J. Raaijmakers J. G. Jonker C. (2003). Similar network activated by young and old adults during the acquisition of a motor sequence. Neurobiol. Aging 24, 1013–1019 10.1016/S0197-4580(03)00030-7 12928061

[B7] Deffains M. Legallet E. Apicella P. (2011). Importance of the temporal structure of movement sequences on the ability of monkeys to use serial order information. Exp. Brain Res. 214, 415–425 10.1007/s00221-011-2839-0 21858500

[B8] Dominey P. F. (1998a). Influences of temporal organization on sequence learning and transfer: comments on Stadler (1995) and Curran and Keele (1993). J. Exp. Psychol. Learn. Mem. Cogn. 24, 14 10.1037/0278-7393.24.1.234

[B9] Dominey P. F. (1998b). A shared system for learning serial and temporal structure of sensori-motor sequences? Evidence from simulation and human experiments. Brain Res. Cogn. Brain Res. 6, 163–172 10.1016/S0926-6410(97)00029-3 9479067

[B10] Dominey P. F. Ramus F. (2000). Neural network processing of natural language: I. Sensitivity to serial, temporal and abstract structure of language in the infant. Lang. Cogn. Process. 15, 40 10.1080/016909600386129

[B11] Doyon J. (2008). Motor sequence learning and movement disorders. Curr. Opin. Neurol. 21, 478–483 10.1097/WCO.0b013e328304b6a3 18607210

[B12] Doyon J. Bellec P. Amsel R. Penhune V. Monchi O. Carrier J. (2009). Contributions of the basal ganglia and functionally related brain structures to motor learning. Behav. Brain Res. 199, 61–75 10.1016/j.bbr.2008.11.012 19061920

[B13] Doyon J. Owen A. M. Petrides M. Sziklas V. Evans A. C. (1996). Functional anatomy of visuomotor skill learning in human subjects examined with positron emission tomography. Eur. J. Neurosci. 8, 637–648 10.1111/j.1460-9568.1996.tb01249.x 9081615

[B14] Doyon J. Ungerleider L. G. (2002). Functional anatomy of motor skill learning, in Neuropsychology of Memory, eds Squire L. R. Schacter D. L. (New York, NY: The Guilford Press), 225–238

[B15] Friston K. J. Holmes A. P. Worsley K. J. Poline J. P. Frith C. D. Frackowiak R. S. J. (1994). Statistical parametric maps in functional imaging: a general linear approach. Hum. Brain Mapp. 2, 189–210 10.1002/hbm.460020402

[B16] Grafton S. T. Hazeltine E. Ivry R. B. (1995). Functional mapping of sequence learning in normal humans. J. Cogn. Neurosci. 7, 497–510 10.1162/jocn.1995.7.4.497 23961907

[B17] Haaland K. Y. Elsinger C. L. Mayer A. R. Durgerian S. Rao S. M. (2004). Motor sequence complexity and performing hand produce differential patterns of hemispheric lateralization. J. Cogn. Neurosci. 16, 621–636 10.1162/089892904323057344 15165352

[B18] Halsband U. Lange R. K. (2006). Motor learning in man: a review of functional and clinical studies. J. Physiol. Paris 99, 414–424 10.1016/j.jphysparis.2006.03.007 16730432

[B19] Harrington D. L. Rao S. M. Haaland K. Y. Bobholz J. A. Mayer A. R. Binderx J. R. (2000). Specialized neural systems underlying representations of sequential movements. J. Cogn. Neurosci. 12, 56–77 10.1162/08989290051137602 10769306

[B20] Hayes A. E. Davidson M. C. Keele S. W. Rafal R. D. (1998). Toward a functional analysis of the basal ganglia. J. Cogn. Neurosci. 10, 178–198 10.1162/089892998562645 9555106

[B21] Hikosaka O. Miyashita K. Miyachi S. Sakai K. Lu X. (1998). Differential roles of the frontal cortex, basal ganglia, and cerebellum in visuomotor sequence learning. Neurobiol. Learn. Mem. 70, 137–149 10.1006/nlme.1998.3844 9753593

[B22] Hikosaka O. Nakamura K. Sakai K. Nakahara H. (2002). Central mechanisms of motor skill learning. Curr. Opin. Neurobiol. 12, 217–222 10.1016/S0959-4388(02)00307-0 12015240

[B23] Jenkins I. H. Jahanshahi M. Jueptner M. Passingham R. E. Brooks D. J. (2000). Self-initiated versus externally triggered movements. II. The effect of movement predictability on regional cerebral blood flow. Brain 123, 1216–1228 10.1093/brain/123.6.1216 10825359

[B24] Jueptner M. Weiller C. (1998). A review of differences between basal ganglia and cerebellar control of movements as revealed by functional imaging studies. Brain 121, 1437–1449 10.1093/brain/121.8.1437 9712006

[B25] Lehericy S. Benali H. Van de Moortele P. F. Pelegrini-Issac M. Waechter T. Ugurbil K. (2005). Distinct basal ganglia territories are engaged in early and advanced motor sequence learning. Proc. Natl. Acad. Sci. U.S.A. 102, 12566–12571 10.1073/pnas.0502762102 16107540 PMC1194910

[B26] Mathiak K. Hertrich I. Grodd W. Ackermann H. (2004). Discrimination of temporal information at the cerebellum: functional magnetic resonance imaging of nonverbal auditory memory. Neuroimage 21, 154–162 10.1016/j.neuroimage.2003.09.036 14741652

[B27] Mauk M. D. Buonomano D. V. (2004). The neural basis of temporal processing. Annu. Rev. Neurosci. 27, 307–340 10.1146/annurev.neuro.27.070203.144247 15217335

[B28] Nichols T. Brett M. Andersson J. Wager T. Poline J. B. (2004). Valid conjunction inference with the minimum statistic. Neuroimage 25, 653–660 10.1016/j.neuroimage.2004.12.005 15808966

[B29] Oldfield R. C. (1971). The assessment and analysis of handedness: the Edinburgh inventory. Neuropsychologia 9, 97–113 10.1016/0028-3932(71)90067-4 5146491

[B30] Penhune V. B. Steele C. J. (2012). Parallel contributions of cerebellar, striatal and M1 mechanisms to motor sequence learning. Behav. Brain Res. 226, 579–591 10.1016/j.bbr.2011.09.044 22004979

[B31] Rauch S. L. Savage C. R. Brown H. D. Curran T. Alpert N. M. Kendrick A. (1995). A PET investigation of implicit and explicit sequence learning. Hum. Brain Mapp. 3, 271–286 10.1002/hbm.46003040311530429

[B32] Rauch S. L. Whalen P. J. Savage C. R. Curran T. Kendrick A. Brown H. D. (1997). Striatal recruitment during an implicit sequence learning task as measured by functional magnetic resonance imaging. Hum. Brain Mapp. 5, 124–132 10.1002/(SICI)1097-0193(1997)5:2<124::AID-HBM6>3.0.CO;2-5 10096417

[B33] Sakai K. Hikosaka O. Miyauchi S. Takino R. Tamada T. Iwata N. K. (1999). Neural representation of a rhythm depends on its interval ratio. J. Neurosci. 19, 10074–10081 10559415 10.1523/JNEUROSCI.19-22-10074.1999 PMC6782989

[B34] Sakai K. Hikosaka O. Nakamura K. (2004). Emergence of rhythm during motor learning. Trends Cogn. Sci. 8, 547–553 10.1016/j.tics.2004.10.005 15556024

[B35] Sakai K. Ramnani N. Passingham R. E. (2002). Learning of sequences of finger movements and timing: frontal lobe and action-oriented representation. J. Neurophysiol. 88, 2035–2046 12364526 10.1152/jn.2002.88.4.2035

[B36] Shin J. C. Ivry R. B. (2003). Spatial and temporal sequence learning in patients with Parkinson's disease or cerebellar lesions. J. Cogn. Neurosci. 15, 1232–1243 10.1162/089892903322598175 14709239

[B37] Stadler M. A. (1993). Implicit serial learning: questions inspired by Hebb (1961). Mem. Cogn. 21, 819–827 10.3758/BF03202749 8289659

[B38] Stadler M. A. (1995). Role of attention in implicit learning. J. Exp. Psychol. Learn. Mem. Cogn. 21, 674–685 10.1037/0278-7393.21.3.674

[B39] Talairach J. Tournoux P. (1988). Co-planar Stereotaxic Atlas of the Human Brain. New York, NY: Thieme

[B40] Ventre-Dominey J. Dominey P. F. Broussolle E. (2002). Dissociable processing of temporal structure in repetitive eye-hand movements in Parkinson's disease. Neuropsychologia 40, 1407–1418 10.1016/S0028-3932(01)00207-X 11931945

[B41] Verwey W. B. (1996). Buffer loading and chunking in sequential keypressing. J. Exp. Psychol. Hum. Percept. Perform. 22, 544–562 10.1037/0096-1523.22.3.544

[B42] Verwey W. B. (1999). Evidence for a multi-stage model of practice in a sequential movement task. J. Exp. Psychol. Hum. Percept. Perform. 25, 1693–1708 10.1037/0096-1523.25.6.1693

[B43] Verwey W. B. (2003). Processing modes and parallel processors in producing familiar keying sequences. Psychol. Res. 67, 106–122 12739146 10.1007/s00426-002-0120-7

[B44] Verwey W. B. Abrahamse E. L. (2012). Distinct modes of executing movement sequences: reacting, associating, and chunking. Acta Psychol. 140, 274–282 10.1016/j.actpsy.2012.05.007 22705631

[B45] Verwey W. B. Abrahamse E. L. (2009). Segmentation of short keying sequences does not spontaneously transfer to other sequences. Hum. Mov. Sci. 28, 348–361 10.1016/j.humov.2008.10.004 19135276

[B46] Verwey W. B. Eikelboom T. (2003). Evidence for lasting sequence segmentation in the discrete sequence-production task. J. Mot. Behav. 35, 171–181 10.1080/00222890309602131 12711587

[B47] Vuust P. Wallentin M. Mouridsen K. Ostergaard L. Roepstorff A. (2011). Tapping polyrhythms in music activates language areas. Neurosci. Lett. 494, 211–216 10.1016/j.neulet.2011.03.015 21397659

